# Roles of TRPM8 Ion Channels in Cancer: Proliferation, Survival, and Invasion

**DOI:** 10.3390/cancers7040882

**Published:** 2015-10-23

**Authors:** Nelson S. Yee

**Affiliations:** 1 Division of Hematology-Oncology, Department of Medicine, Penn State College of Medicine, Pennsylvania State University, Hershey, PA 17033, USA; 2 Program of Experimental Therapeutics, Penn State Hershey Cancer Institute, Pennsylvania State University, Hershey, PA 17033, USA; 3 Penn State Milton S. Hershey Medical Center, Pennsylvania State University, Hershey, PA 17033, USA; nyee@hmc.psu.edu; Tel.: +1-717-531-0003 (ext. 289532); Fax: +1-717-531-5076

**Keywords:** calcium, cancer, cell cycle, ion channels, invasion, proliferation, senescence, survival, TRPM8

## Abstract

The goal of this article is to provide a critical review of the transient receptor potential melastatin-subfamily member 8 (TRPM8) in cancers, with an emphasis on its roles in cellular proliferation, survival, and invasion. The TRPM8 ion channels regulate Ca^2+^ homeostasis and function as a cellular sensor and transducer of cold temperature. Accumulating evidence has demonstrated that TRPM8 is aberrantly expressed in a variety of malignant solid tumors. Clinicopathological analysis has shown that over-expression of TRPM8 correlates with tumor progression. Experimental data have revealed important roles of TRPM8 channels in cancer cells proliferation, survival, and invasion, which appear to be dependent on the cancer type. Recent reports have begun to reveal the signaling mechanisms that mediate the biological roles of TRPM8 in tumor growth and metastasis. Determining the mechanistic roles of TRPM8 in cancer is expected to elucidate the impact of thermal and chemical stimuli on the formation and progression of neoplasms. Translational research and clinical investigation of TRPM8 in malignant diseases will help exploit these ion channels as molecular biomarkers and therapeutic targets for developing precision cancer medicine.

## 1. Introduction

This article focuses on the transient receptor potential melastatin-subfamily member 8 (TRPM8) ion channel in cancers, with an emphasis on its roles in proliferation, survival, and invasion. As a regulator of Ca^2+^ homeostasis, the TRPM8 ion channel functions as a cellular sensor and transducer of cold temperature. While normal expression is tissue-selective, TRPM8 is aberrantly expressed in a variety of malignant tumors. Experimental data implicate that TRPM8 channels play important roles in cancer cells proliferation, survival, migration, invasion, and neurosecretion. Several *in vivo* studies have implicated the contributory roles of TRPM8 in cancer growth and metastasis. The etiological role of TRPM8 as a cold sensor in malignant neoplasia is essentially unknown. Research efforts to determine the mechanistic roles of TRPM8 in cancer are expected to shed new light on how the physical and chemical alterations in the environment impact on carcinogenesis.

TRPM8 is a member of the TRP superfamily of ion channels that normally function in diverse physiological responses [1]. The human *TRPM8* gene in chromosome two encodes a non-selective, voltage-gated, and Ca^2+^ permeable ion channel [2,3]. TRPM8 protein consists of 1104 amino acid residues, with a molecular mass of 128 kDa. In human adult tissues, *TRPM8* mRNA is highly expressed in the prostate gland [4]. It is also detected in the liver, dorsal root ganglion, and trigeminal ganglion neurons [5]. Under physiological conditions, TRPM8 ion channels are necessary for sensation of coolness [6,7,8] and serum homeostasis of insulin [9]. Accumulating evidence implicates that TRPM8 is involved in diverse human disorders, particularly cancer.

In this article, I provide an overview of the TRPM8 ion channel regarding its structural features and physiological functions. The expression and roles of TRPM8 channels in various cancers will be described, with an emphasis on cancer type-dependent cellular proliferation, survival, and invasion. How TRPM8 may contribute to cancer growth and metastasis as well as the clinical significance of TRPM8 in malignant tumors will be discussed. I hope this article will help stimulate research efforts and collaboration to understand the mechanistic roles of TRPM8 in malignant neoplasia, and to explore the potential of TRPM8 as a molecular biomarker and therapeutic target in precision oncology.

## 2. Structure and Functions of TRPM8 Ion Channels

**Figure 1:**
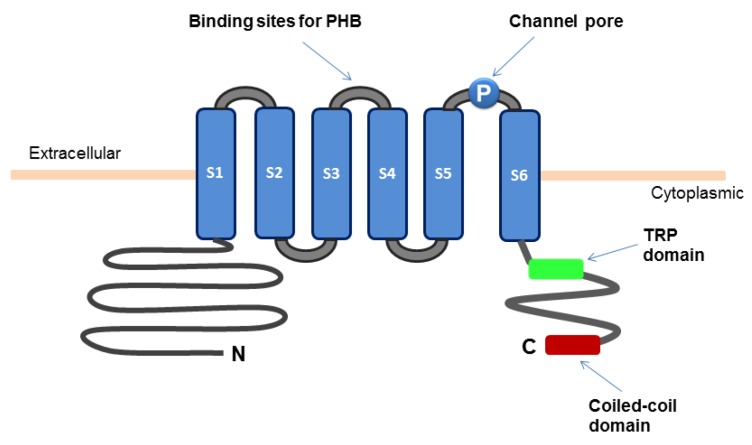
Schematic diagram for the structure of TRPM8 ion channel.

The TRPM8 channel is composed of six transmembrane segments (S1–S6) and the intracellular amino and carboxyl termini (Figure 1). The S2 and S3 segments contain the binding sites for menthol and icilin, respectively. The S4 segment and the region between S4 and S5 possess the function of voltage sensing. The region between S5 and S6 forms the channel pore [10]. Functional TRPM8 channels are tetramers, each composed of four TRPM8 subunits [11,12,13,14]. The coiled-coil domain at the carboxyl terminus of TRPM8 protein has been implicated in its oligomerization [15,16]. The TRP domain also located within the carboxyl terminus is important for temperature-dependent channel opening and for channel activation by phosphatidylinositol 4,5-bisphosphate (PIP_2_) and menthol [17,18,19]. A segment in the amino-terminus is involved in localization of TRPM8 protein to plasma membrane and its stability [12,16]. The serine and threonine residues (S9 and T17, respectively) in the amino terminus represent phosphorylation sites of protein kinase A (PKA) that negatively regulates the TRPM8 channel activity [20]. The glycosylated Asn934 near the channel pore and the surrounding Cys929 and Cys940 are important for channel activity. Recent evidence indicates that the TRPM8 channel forms a structural complex with poly-(*R*)-3-hydroxybutyrate (PHB) [21]. Covalent binding of poly-(*R*)-3-hydroxybutyrate (PHB) to the serine residues in the linker between S3 and S4 are involved in TRPM8-mediated response to cooling or menthol. Moreover, splice variants of TRPM8 with alteration of the amino terminus have been identified [22]. Some of the isoforms act as dominant negative regulators of the TRPM8 channel activity [23,24].

The TRPM8 channel can be activated at temperature between 15 °C and 25 °C, resulting in a transient rise in the intracellular level of Ca^2+^ [Ca^2+^]_ic_ [2,3,6,7,8]. Alternatively, addition of cooling agents, such as menthol, eucalyptol, and icilin, stimulates the activity of TRPM8 channels. Distinct mechanisms are involved in each of these modes of activation of TRPM8. Opening of the TRPM8 channel is voltage-dependent and facilitated with membrane depolarization, and the depolarization potential for channel activation is reduced by thermal cooling [25]. Menthol directly stimulates the TRPM8 activity by shifting the voltage dependence towards a more negative potential and also by shifting the response-threshold temperature of TRPM8 towards warmer temperature [26]. Menthol-induced activation of the TRPM8 channel involves the residues on S2 and its carboxyl terminus [19]. Icilin-mediated stimulation of the TRPM8 channel activity requires the presence of extracellular Ca^2+^ [27].

The TRPM8 channel activity can be modulated by pH, PIP_2_, and endogenous signaling molecules_._ Increase in extracellular acidity (from pH 7.3 to 6) abolished the channel activity in response to cold stimulation or icilin, but not menthol [28]. In the range of extracellular pH 8.1–6.5, the temperature threshold for channel activation is raised at higher pH but reduced at lower pH [28]. Intracellular acidification lowers the threshold for activation by coolness and diminishes the amplitude of icilin-induced current [28]. However, activation of TRPM8 by cold temperature and cooling compounds requires PIP_2_ at the plasma membrane [17,18]. Furthermore, PIP_2_ interacts with the positive residues of the carboxyl terminus in TRPM8, and the affinity of PIP_2_ for TRPM8 is increased by coolness. As a negative feedback mechanism, the TRPM8-mediated Ca^2+^ influx activates Ca^2+^-sensitive phospholipase C that hydrolyzes PIP_2_ to diacylglycerol, which further inhibits TRPM8 via activation of PKC-mediated dephosphorylation of TRPM8 [17,29]. On the other hand, activators of the PKA pathway (8-Br-cAMP and forkoslin) and the endogenous cannabinoids/vanilloids (anandamide and *N*-arachidonoyl-dopamine) as well as stimulation of G_i_-coupled α2A-adrenoreceptor inhibit the TRPM8-mediated nociception of coolness [20,30]. Additionally, the prostate kallikrein, prostate-specific antigen (PSA), increases expression of TRPM8 channels on the plasma membrane and enhances coolness-induced TRPM8-mediated current through the bradykinin 2 receptor signaling pathway [31]. These data suggest that PSA is a physiological agonist of TRPM8. In recent studies, the TRP channel-associated factors (TCAF1 and TCAF2) have been identified as binding partners of TRPM8 channel [32]. It has been demonstrated that the TCAFs can regulate trafficking of TRPM8 to the cell surface as well as gating of the TRPM8 channels.

Recent findings have shown that TRPM8 protein is a testosterone receptor, and androgen response element mediates androgen regulation of the *TRPM8* gene [33,34,35]. These studies further demonstrated that testosterone directly binds to the TRPM8 protein and activates TRPM8-mediated currents and Ca^2+^ responses [33]. Moreover, testosterone applied at picomolar concentrations induces full opening of the TRPM8 channels and a cooling sensation in human skin [34]. These data suggest that testosterone plays a regulatory role in the TRPM8 channel function, and imply that TRPM8 channels are involved in testosterone-dependent physiological processes.

Thus, the TRPM8 channel activity can be influenced by physical and chemical alterations in the microenvironment, whereas PIP_2_, changes in pH, PKC/PKA signaling, PSA, and TCAFs modulate the response of TRPM8 to cold temperature and cooling agents. In addition, the data demonstrating functional interaction between TRPM8 protein and testosterone are important for understanding the physiological functions of TRPM8 and testosterone-mediated behavioral traits. It may also provide clues to how aberrant expression and activity of TRPM8 channels contribute to the pathogenesis of human diseases particularly cancer. In the following section, the expression of TRPM8 in malignant neoplasms is described. The various roles of TRPM8 in cancer including proliferation, survival, and invasion are reviewed.

## 3. TRPM8 Channels in Cancers

### 3.1. Expression of TRPM8 Ion Channels in Cancers

Accumulating studies have demonstrated that TRPM8 is over-expressed in a variety of human neoplastic tissues and cell lines. These findings are based on immunohistochemical analysis of TRPM8 using specific antibodies, *in situ* hybridization using riboprobes, and also quantitative polymerase chain reactions (PCR). Evidence to date indicates that TRPM8 is expressed in a variety of solid tumors, and the functional roles of TRPM8 channels in cancer cells have been identified (Table 1). The clinical significance for the expression of TRPM8 has been further studied in some of the malignant diseases.

**Table 1:** Expression and functional roles of TRPM8 in human cancers.

Cancer	Expression	Functional Role	References
Prostatic carcinoma	Up-regulated in tissues and androgen receptor-expressing cell lines (LNCaP, VcaP, C4-2B, NCI-H660).	Cell proliferation, survival, migration, hypoxic growth, xenograft growth, angiogenesis	[31,32,35,36,37,38,39,40,41,42,43,44,45,46]
Pancreatic carcinoma	Up-regulated in cell lines (PL45, MIA PaCa-2, PANC-1, HPAF-II, BxPC-3, Capan-1, Panc 02.03). Over-expressed in pancreatic adenocarcinoma. Also aberrantly expressed in chronic pancreatitis, pancreatic intraepithelial neoplasm, intraductal papillary mucinous neoplasm, solid pseudopapillary neoplasm, adenosquamous carcinoma, and neuroendocrine tumor.	Cell proliferation, cell cycle progression, replicative senescence, survival, migration, invasion.	[47,48,49,50,51]
Breast adenocarcinoma	Over-expressed in cell line (MCF-7, T47D, MDA-MB231, BT549, SKBR3, ZR-75-30). Over-expressed in breast adenocarcinoma tissues.	Cell migration, invasion	[40,52,53,54]
Lung carcinoma	Expressed in tissues and cell lines (LLC-1, LLC-2, LLC-3).	Cell proliferation, adhesion, migration, invasion, resistance to hypothermia.	[40,55]
Colorectal adenocarcinoma	Expressed in tissues and cell lines (Caco-2, HCT 116).	Cell growth, survival, xenograft tumor growth, chemically-induced cancer growth.	[40,56]
Melanoma	Expressed in tissues and cell lines (G-361, A-375, Mel 202, Mel 270, 92.1, omm 2.3).	Cell survival	[40,57,58,59]
Urinary bladder carcinoma	Expressed in cell line (T24). Over-expressed in urothelial carcinoma tissues.	Cell survival	[60,61]
Neuroblastoma	Up-regulated expression in cell line (IMR-32) in response to 5-bromo-2-deoxyuridine induced differentiation.	Not reported	[62]
Glioblastoma multiforme	Expressed in cell line (DBTRG) and tissues.	Cell migration, survival	[63,64]
Neuroendocrine tumor	Expressed in neuroendocrine tumor cell line (BON) and tissues.	Secretion of neurotensin.	[50,65]
Oral squamous cell carcinoma	Expressed in cell lines derived from tongue (HSC3 and HSC4).	Cell migration and invasion.	[66]
Osteosarcoma	Expression in osteosarcoma cell lines (U2OS, MG-63, SaOS2, HOS); increased expression in osteosarcoma as compared to osteochondroma.	Cell proliferation, cell cycle progression, survival, migration, and invasion.	[67]

The expression and functional significance of TRPM8 have been examined in genitourinary carcinoma (Table 1). In the prostate gland, expression of TRPM8 requires androgen and its receptor, and sub-cellular localization of TRPM8 channels appears to depend on the status of cellular differentiation [36,37,38,39]. This is consistent with the recent finding that androgen response element mediates androgen regulation of the *TRPM8* gene [35]. TRPM8 protein is expressed in the plasma membrane of differentiated secretory prostate epithelia and primary tumor of prostate gland, but not in the undifferentiated basal cells. On the other hand, expression of TRPM8 in the endoplasmic reticulum is independent of the differentiation status of prostate cells. The functional significance of TRPM8 in prostate epithelia has been revealed by the experiments using electrophysiological analysis and Ca^2+^ measurement. Stimulation of prostate cancer cells (LNCaP) by either coolness, menthol, or icilin induced a membrane current characterized by inward rectification and high Ca^2+^ selectivity [38]. This membrane current involves Ca^2+^ release from endoplasmic reticulum and concomitant Ca^2+^ influx through activation of store-operated channels in plasma membrane.

In prostate tumor tissues, TRPM8 mRNA is over-expressed relative to non-tumor tissues [40,41]. Increased immunoreactivity to anti-TRPM8 antibodies was demonstrated in hormone-refractory prostate cancer tissues and in tumors with higher Gleason scores [42]. In addition, the TRPM8 mRNA levels in the urine and blood of patients with metastatic prostate tumors are significantly elevated as compared to healthy individuals, but the increase is not significantly different from those with localized disease [43]. Recent evidence indicates that TRPM8 protein undergoes ubiquitin-mediated degradation in prostate cancer cells, and the TRPM8 channel activity on the plasma membrane could be increased by inhibiting the initial enzyme in ubiquitination [35]. However, findings from the expression analyses suggest that TRPM8 channels play a regulatory role in prostate cancer growth and metastasis.

Besides prostate carcinoma, the expression levels of TRPM8 were significantly higher in urothelial carcinoma of bladder tissues than in non-cancerous urothelial tissues [60]. A positive association between the expression levels of TRPM8 and histological grade or tumor stage was established. Moreover, high expression of TRPM8 was shown to correlate with poor survival of patients with urothelial carcinoma of urinary bladder.

The expression pattern and levels of TRPM8 in pre-malignant pancreatic tissues and various subtypes of pancreatic neoplasms have been investigated [47,48,49,50]. Initial studies demonstrated that TRPM8 is over-expressed in pancreatic adenocarcinoma cell lines and tissues, as compared to non-cancerous pancreatic ductal epithelia and tissues [47]. In normal pancreatic tissue, anti-TRPM8 immunoreactivity can be detected in the ductal epithelia, centroacinar cells, and islet endocrine cells. TRPM8 is also aberrantly over-expressed in chronic pancreatitis, pancreatic intra-epithelial neoplasms, and intraductal papillary mucinous neoplasms, and various malignant tumors (Table 1). Immunohistochemical analysis demonstrates that TRPM8 is expressed at either moderate or high levels in the majority of pancreatic adenocarcinoma specimens. Statistical analysis indicates that the aberrant over-expression of TRPM8 in pancreatic adenocarcinoma significantly correlates with tumor size and tumor stages [50].

Expression of TRPM8 has also been identified in other malignancies such as lung carcinoma, colorectal melanoma, glioblastoma multiforme, neuroendocrine tumor, and oral squamous cell carcinoma (Table 1). In particular, TRPM8 has been found to be over-expressed in breast carcinoma, neuroblastoma, and osteosarcoma, as compared with the corresponding normal tissues (Table 1). Moreover, expression of TRPM8 in breast carcinoma correlates with histological grade, Ki-67, tumor size, and expression of estrogen receptor. These findings suggest that TRPM8 channels play a role in the development and growth of mammary tumor [52,53,54]. The clinical significance of TRPM8 channels in these malignant tumors remains to be demonstrated.

To date, expression of TRPM8 in hematological malignancies has not been reported. In prostate and breast carcinoma, expression of TRPM8 is regulated by androgen and estrogen, respectively [36,53]. Little is known about the mechanism underlying the up-regulated expression of TRPM8 in the other malignant tumors. Analysis of genomic DNA in pancreatic adenocarcinoma cell lines by real-time PCR suggests that amplification of *TRPM8* DNA is unlikely to be involved [50]. However, functional studies have begun to reveal important roles of TRPM8 ion channels in neoplasia.

### 3.2. Roles of TRPM8 Ion Channels in Cancers

Emerging studies have demonstrated that TRPM8 channels are involved in cellular proliferation, survival, and invasion—some of the hallmarks of cancer. Current evidence suggests that TRPM8 channels play contributory roles in tumor growth and metastasis. Results of the studies thus far show that TRPM8 can have opposing effects on cancer cells proliferation, survival, and invasion. Such discrepancy may depend on the type of cancer cells, their molecular phenotypes, and the interventions by which expression and activity of TRPM8 channels are modulated. However, correlation of the expression levels of TRPM8 in tumors with their clinicopathological features has implicated the clinical significance of TRPM8 channels in malignant diseases. Recent data have begun to reveal the signaling mechanisms underlying the TRPM8 channels-mediated biological effects of cancer.

#### 3.2.1. Role of TRPM8 in Cancer Cells Proliferation

Experimental data support an important role of TRPM8 channels in proliferation of cancer cells (Table 1). These studies were conducted in various types of cancer cell lines including pancreatic, prostatic, pulmonary, and colonic carcinoma, as well as osteosarcoma. The role of TRPM8 in cancer cell proliferation was determined by genetic silencing of TRPM8 expression, ectopic expression of TRPM8, and chemical activation or inhibition of TRPM8 channel activity. Cellular proliferation was evaluated by *in vitro* assays based on hydrolysis of MTS or MTT, by counting cells, and flow cytometric analysis of the cell cycle. The results thus far indicate that TRPM8 plays an important role in regulating the proliferative capability of the cancer cells.

In the pancreatic adenocarcinoma cell lines, BxPC-3 and PANC-1, small interfering RNA (siRNA)-mediated silencing of *TRPM8* reduced cellular proliferation, as determined by MTS assay and counting cells [47]. Consistent with its proliferative role, pancreatic cancer cells transfected with anti-*TRPM8* siRNA exhibited impairment of cell cycle progression [47]. As a result, the cells became arrested in the G_1_ phase and the proportion of cells entering the S phase decreased. These events were associated with accumulation of the cyclin-dependent kinases p21^CDKN2A^ and p27^CDKN2B^, consistent with cell cycle arrest in the G_1_ phase [47].

**Figure 2:**
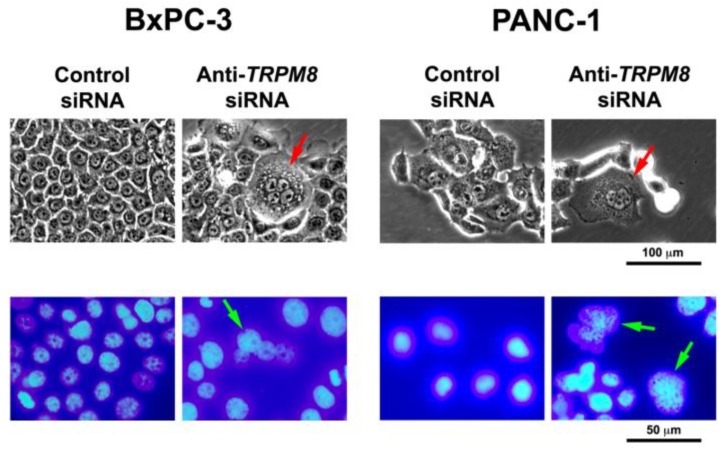
Targeted silencing of *TRPM8* induces mitotic abnormalities and replicative arrest in pancreatic cancer cells. The BxPC-3 and PANC-1 cells were transfected with anti-*TRPM8* siRNA or non-targeting control siRNA and incubated at 37 °C until analysis. Top panel, phase-contrast micrographs showing that TRPM8-deficient cells contain multiple nuclei and cytoplasmic vacuoles. Bottom panel, DAPI-stained fluorescent micrographs showing that TRPM8-deficient cells contain nuclei being arrested in division consistent with multiple nuclei. For comparison, in both phase-contrast and fluorescent micrographs, control siRNA-transfected cells contain round to oval shaped nuclei with a smooth surface, and no or few cytoplasmic vacuoles.

Consistent with the proliferative role of TRPM8, pancreatic cancer cells with down-regulated expression of TRPM8 exhibited features of replicative senescence. Morphological examination revealed the presence of multiple nuclei, suggesting a defect in cell division [49] (Figure 2). Using senescence-associated β-galactosidase (SAβG) as a marker of cellular senescence, siRNA-mediated silencing of TRPM8 induced expression of SAβG [49]. These findings indicate that TRPM8 is required for maintaining the uncontrolled proliferation of cancer cells through regulation of cell cycle progression and replicative senescence.

The proliferative role of TRPM8 in cancer cells is also demonstrated in AR+ prostatic carcinoma (LNCaP), osteosarcoma (MG-63 and U2OS), and colon cancer (Caco-2) cell lines. In the AR+ prostatic carcinoma and osteosarcoma cell lines, anti-*TRPM8* siRNA reduced cellular proliferation and impaired cell cycle progression with arrest in the G_0_/G_1_ phases [44,67]. Consistent with these data, chemical blockers of TRPM8 inhibited proliferation in prostate cancer cells, whereas activator of TRPM8 (such as menthol) stimulated proliferation [44]. The proliferative role of TRPM8 in prostate cancer is further supported by TRPM8-mediated promotion of tumor growth under hypoxic condition *in vitro* [42]. In agreement with these findings, cannabigerol (derived from *Cannabis*) has been shown to block TRPM8 channel and inhibit colon cancer cell growth [56].

On the other hand, over-expression of TRPM8 produces anti-proliferative effect in certain type of cancer cells. In AR- prostate cancer PC-3 cells that do not express TRPM8, ectopic expression of TRPM8 showed inhibitory effect on cellular proliferation [45]. Notably, genetic or chemical inhibition of TRPM8 has minimal, if any, effect on proliferation in non-tumoral prostate cell line (PNT1A). Consistent with the anti-proliferative role of TRPM8 in PC-3 cells, siRNA-mediated down-regulation of TRPM8 promoted cell proliferation of lung cancer cells LLC-2 [55].

Taken together, the data from those experiments using different types of cancer cells support an important role of TRPM8 in cellular proliferation that is dependent on the cellular, and probably the molecular, context. In the cell lines derived from pancreatic adenocarcinoma, AR+ prostatic carcinoma, osteosarcoma, and colon cancer, TRPM8 is required for sustaining cellular proliferation. In AR− prostatic carcinoma and the lung cancer cell line, TRPM8 channels play a negative role in cellular proliferation. It will be interesting to understand how the cancer cell types as well as the molecular phenotypes influence the roles of TRPM8 in proliferation. However, results of the studies thus far suggest a cell-type dependent modulatory role of TRPM8 in tumor growth and progression through its regulatory effects on cancer cells proliferation. This notion is further demonstrated by the *in vivo* studies showing that over-expression of TRPM8 in the AR+ and TRPM8-expressing LNPaC cells promoted tumorigenicity of xenograft prostate cancer [42]. On the other hand, ectopic expression of TRPM8 in the AR− and TRPM8-non-expressing PC-3 cells produced a negative effect on the growth and progression of xenograft prostate tumor [46]. Moreover, the TRPM8 channel blocker, cannabigerol, inhibited the growth of xenograft colon tumor (HCT 116), as well as chemically-induced colon cancer (azoxymethane model) [56].

#### 3.2.2. Role of TRPM8 in Survival of Cancer Cells

The role of TRPM8 in survival of cancer cells has been examined in several types of tumors (Table 1). In the AR+ prostate cancer cell line LNCaP, siRNA-mediated knockdown of TRPM8 or using a chemical blocker of TRPM8 (capsazepine) reduced cell viability (by MTT assay) and induced apoptotic nuclei [36]. Similarly, the *Cannabis* derivative cannabigerol with blocking activity of the TRPM8 channel induced apoptosis in colon cancer cells [56]. However, in pancreatic adenocarcinoma cell lines (BxPC-3 and PANC-1), siRNA-mediated down-regulation of TRPM8 did not induce apoptotic cell death as determined by flow cytometric analysis [49].

On the other hand, using menthol to activate the TRPM8 channel, the cell viability was decreased as determined by MTT assay, cell morphology, and PrestoBlue^®^ assay. The menthol-induced reduction of cell viability was observed in the cell lines derived from melanoma (G-361, A-375) and urinary bladder carcinoma (T24) [57,58,59,60,61]. The pro-death effect of menthol might be due to a sustained elevation of [Ca^2+^]_ic_ or an off-target effect. Consistent with this finding, addition of testosterone (agonist of TRPM8 channel) or PYR-41 (inhibitor of ubiquitin-mediated degradation of TRPM8 protein) increased activity of TRPM8 in prostate cancer cells, leading to Ca^2+^ influx and apoptotic cell death [35]. Thus, the role of TRPM8 in cell survival and apoptosis appears to depend on the cancer cell types and how the TRPM8 expression/activity is modulated.

#### 3.2.3. Role of TRPM8 in Cancer Cells Migration and Invasion

The effects of modulating the expression and activity of TRPM8 channels in cancer cells migration and invasion have been investigated (Table 1). In glioblastoma cells, addition of menthol stimulates an increase in [Ca^2+^]_ic_ and their ability of migration, presumably by activating TRPM8 [63]. Consistent with its pro-migratory role, menthol enhances the ability of cell migration and invasion by potentiating MMP-9 activity in oral squamous cell carcinoma; these effects were suppressed by the TRPM8 antagonist RQ-00203078 [66]. The ability of invasion in pancreatic cancer cells was investigated in transwell inserts coated with a solubilized tumor-associated basement membrane matrix. Pancreatic adenocarcinoma cell lines (BxPC-3 and MIA PaCa-2) incubated with short hairpin RNA (shRNA)-mediated silencing of *TRPM8* demonstrated reduced their ability to invade [50]. Similarly, anti-*TRPM8* siRNA decreased the ability of cell adhesion and invasion in lung cancer and osteosarcoma cells [55,67]. Consistent with these findings, the pro-migratory and pro-invasive roles of TRPM8 channels were demonstrated in breast cancer cells by ectopically modulating the expression of TRPM8 [54]. Furthermore, these cellular effects were associated with changes in the levels of E-cadherin, fibronectin, vimentin, and SNAIL [54]. Results of these studies support important roles of TRPM8 channels in epithelial-mesenchymal transformation and tumor metastasis.

On the contrary, ectopic expression of TRPM8 in AR− prostate cancer cells impaired cell migration through inactivation of focal adhesion kinase [45]. Consistent with this finding, in human embryonic kidney cells or AR− prostate cancer cells ectopically expressing TRPM8, cellular motility was reduced by PSA and/or icilin that increased stimulated TRPM8 channel activity and expression [31]. In agreement with this, TCAF1 that facilitates opening of the TRPM8 channel has been demonstrated to impede prostate cancer cells migration [32]. Similarly, in pancreatic cancer cells, siRNA-mediated silencing of TRPM8 enhanced migration, while activation of TRPM8 inhibited migration [51]. These data indicate that the roles of TRPM8 in cancer cells migration and invasion may depend on the cellular context and the intervention by which TRPM8 expression/activity is modulated. However, these studies implicate that TRPM8 channels are involved in tumor metastasis, though the precise roles remain to be clarified.

#### 3.2.4. Mechanisms of TRPM8-Mediated Biological Processes in Cancer

Recent studies have begun to reveal the mechanisms that mediate the various roles of TRPM8 channels in cancer cells proliferation, survival, migration, and invasion. Electrophysiological and biochemical studies in different types of cells have provided clues regarding the potential signaling mechanisms that mediate the various cellular responses of TRPM8 channels. TRPM8-mediated currents and the associated increase in [Ca^2+^]_ic_ have been demonstrated in various types of cancer cells. Hypothetically, the transient alteration of [Ca^2+^]_ic_ leads to modulation of the signaling pathways and transcription of genes that mediate the cellular responses to mitogens and chemoattractants.

For instance, TRPM8-mediated proliferation, migration, and invasion in osteosarcoma cells are associated with activation of AKT-GSK-3β and phosphorylation of extracellular growth factor-regulated kinase (ERK) and focal adhesion kinase (FAK) [67]. Similarly, TRPM8-promoted cell migration and invasion in breast cancer cells are associated with phosphorylation of AKT and GSK-3β, as well as changes in the levels of E-cadherin, fibronectin, vimentin, and SNAIL [54]. In a recent report, TRPM8-promoted hypoxic tumor growth in AR+ prostate carcinoma cells involves RACK1 binding to HIF-1α and RACK1-mediated ubiquitination of HIF-1α [42].

On the other hand, the anti-tumor effect of ectopically expressing TRPM8 in AR- prostate cancer xenograft is associated with decreased tumor neovascularization [46]. The reduced microvascular density is accompanied with down-regulated expression of vascular endothelial growth factor and phosphorylated FAK [46]. Moreover, the anti-proliferative and pro-apoptotic roles of TRPM8 in prostate cancer cells involve activation of p53 and caspase-9 [35]. Furthermore, putative binding sites for p53 were found in the TRPM8 promoter, and over-expression of p53 up-regulates expression of TRPM8 mRNA [35]. This finding suggests that *TRPM8* is a target gene of p53, which mediates testosterone induced apoptotic cell death in prostate cancer through activation of TRPM8 channels and induced Ca^2+^ uptake.

Increasing data are expected to reveal the signaling mechanisms that mediate the various roles of TRPM8 channels in cancer cells proliferation, survival, migration, and invasion. Tentatively, the signaling pathways downstream of TRPM8 channels are likely dependent on the cancer cells type and their genetic milieu. However, experimental studies in a defined cellular and molecular context may help shed light on the mechanistic roles of TRPM8 in cancer biology.

## 4. Conclusions and Future Perspectives

Accumulating evidence has revealed the aberrant expression and biological roles of the TRPM8 channels in various human malignant tumors. These include cellular proliferation through control of cell cycle progression and replicative senescence, survival, migration, and invasion. In agreement with these cellular functions of TRPM8, the data from *in vivo* studies and clinicopathological correlation suggest important roles of TRPM8 channels in cancer growth and metastasis. Recent reports have begun to elucidate the signaling mechanisms that mediate the various biological roles of TRPM8 in cancer cells. The relationship between TRPM8-mediated sensation and transduction of cold temperature and malignant neoplasia remains to be explored. These areas of TRPM8 in physiology and cancer will be important foci of future investigation. Results of those studies are expected to shed new lights on the molecular mechanisms underlying carcinogenesis, and generate new hypotheses regarding the influence of temperature on neoplasia. Moreover, the aberrant over-expression of TRPM8 in malignant tissues, as well as its proliferative and invasive roles, suggest a unique opportunity for development of TRPM8 channel as a prognostic/predictive biomarker and a therapeutic target in precision oncology.
